# Perceived stress, social support, and insomnia in hemodialysis patients and their family caregivers: an actor-partner interdependence mediation model analysis

**DOI:** 10.3389/fpsyg.2023.1172350

**Published:** 2023-06-29

**Authors:** Yuxiu Tao, Tongcun Liu, Kaipeng Zhuang, Lijuan Fan, Yan Hua, Chunping Ni

**Affiliations:** ^1^School of Nursing, Air Force Medical University, Xi’an, China; ^2^Department of Joint Surgery, The 940th Hospital of PLA Joint Logistics Support Force, Lanzhou, China; ^3^Blood Purification Center, The 940th Hospital of PLA Joint Logistics Support Force, Lanzhou, China

**Keywords:** perceived stress, insomnia, social support, hemodialysis, actor-partner interdependence mediation model

## Abstract

**Objectives:**

Patients’ and caregivers’ physical and mental health may interact. The theory of dyadic illness management holds that patients and caregivers should be regarded as a whole in health management. Previous studies have found that hemodialysis patients and their family caregivers experience poor sleep quality. Perceived stress and social support have effects on insomnia. However, the dyadic interaction between perceived stress, social support, and insomnia among hemodialysis patients and caregivers is unclear. This study aimed to explore the mediating role of social support in the association between perceived stress and insomnia in hemodialysis patient-caregiver dyads.

**Methods:**

A total of 259 hemodialysis patient-caregiver dyads completed the Chinese Perceived Stress Scales (CPSS), the Perceived Social Support Scale (PSSS), and the Athens Insomnia Scale (AIS) in Lanzhou, China, from May 2022 to July 2022. The actor-partner interdependence mediation model analysis was used for data analysis.

**Results:**

In the actor effect, there was a significant positive correlation between perceived stress and insomnia in hemodialysis patients (*β* = 0.091, *p* = 0.001) and their family caregivers (*β* = 0.588, *p* < 0.001). Patient’s and caregiver’s social support played partial mediating roles in the relationship between caregiver’s perceived stress and insomnia (*β* = 0.135, *p* < 0.001 and *β* = 0.111, *p* < 0.001). In the partner effect, caregiver’s perceived stress was positively connected with patient’s insomnia (*β* = 0.915, *p* < 0.001), and the mediating effect of patient’s social support on the relationship between caregiver’s perceived stress and patient’s insomnia was statistically significant (*β* = −0.040, *p* = 0.046).

**Conclusion:**

The perceived stress, social support and insomnia of hemodialysis patients and their family caregivers had interactive effects. Effective dyadic-based interventions should be developed to improve hemodialysis patients’ and caregivers’ sleep quality.

## Introduction

Hemodialysis (HD) is one of the most common treatments for end-stage renal disease (ESRD) patients, which can cause a variety of complications, such as sleep disorders (Hejazian et al., 2021). A meta-analysis showed that 46% of hemodialysis patients experienced insomnia (Tan et al., 2022). Insomnia can reduce quality of life and increase mortality in hemodialysis patients (Orasan et al., 2020). Hemodialysis patients need to be treated regularly for a long time. Their family caregivers play an important role in their treatment and lives. The dyadic illness management theory considers patients and caregivers as a whole and emphasizes disease management as a dualistic phenomenon (Lyons and Lee, 2018). Caregivers bear a great burden of care, which not only affect their quality of life but also impact hemodialysis patient’s outcomes (Sajadi et al., 2020). Studies showed that there was a considerable proportion of caregivers of hemodialysis patients had high levels of psychological problems (Gerogianni et al., 2021) and were at a high risk of sleep disorders (Intas et al., 2020). Meanwhile, caregivers’ psychological problems can affect hemodialysis patients’ psychological problems and hospitalization (Fu et al., 2022). Therefore, it is critical to comprehensively consider the sleep quality of hemodialysis patients and their family caregivers.

Perceived stress refers to the confusion or uncertainty that an individual feels when assessing the threat of a stressful event (Cohen et al., 1983). Because of the disease symptoms and treatment, hemodialysis patients have great perceived stress. This perceived stress may lead to decreased quality of life, increased hospitalization, and mortality (Nadort et al., 2022). Because of the patient’s condition, long-term treatment, and psychological status, informal caregivers experience stress, lowing their quality of life (Campbell, 1998). The conservation of resources theory (COR) suggests that people’s coping resources are related to how they evaluate stress (Hobfoll, 1989), and such assessment, in turn, affects their health outcomes. Many empirical studies have found that perceived stress significantly negatively predicts sleep quality (Eskildsen et al., 2017; Tselebis et al., 2020). Reducing perceived stress is extremely important in improving hemodialysis patients’ and caregivers’ health.

The stress-buffering model suggests that when individuals perceive stress, social support can inhibit the adverse effects of stress (Cohen and Wills, 1985). The severity of insomnia was found to be significantly related to low levels of social support (Kim and Suh, 2019). Increasing social support can help to mitigate the negative effects of insomnia (Chang et al., 2022). Furthermore, higher perceived stress was associated with lower social support (Moseley et al., 2021). Research showed that there was a relationship between perceived stress, sleep quality, and social support among pregnant women (Alan et al., 2020). However, few studies explored the connection between perceived stress, social support, and insomnia in hemodialysis patient-caregiver dyads.

Based on the theories and findings of the preceding studies, the hypotheses of this study were: (1) perceived stress and social support were connected with insomnia both in hemodialysis patients and their family caregivers; (2) social support mediated the relationship between perceived stress and insomnia in hemodialysis patient-caregiver dyads.

## Method

### Study design

A cross-sectional study was conducted to explore the relationship between perceived stress, social support, and insomnia in hemodialysis patient-caregiver dyads.

### Participants

Participants were recruited by convenience sampling from two blood purification centers of two tertiary hospitals from May 2022 to July 2022. The two hospitals are both located in Lanzhou, China. There are 230 patients on long-term hemodialysis treatment in one of the blood purification centers, and 207 in another. There are professional doctors and nurses in the blood purification centers to serve dialysis patients. Most hemodialysis patients receive hemodialysis treatment 3 times per week or 5 times every 2 weeks for about 4 h each time. The etiology of ESRD is mostly primary chronic glomerulonephritis, diabetic nephropathy, and hypertensive nephropathy. Data collection began after obtaining departmental approval.

The inclusion criteria for patients were: (1) aged 18 years or older; (2) received regular hemodialysis treatment for at least 3 months; (3) were conscious and able to cooperate to complete the investigation. Exclusion criteria were: (1) with significant cognitive or mental impairments; (2) had serious heart disease, cerebrovascular accident, or other diseases. The inclusion criteria for caregivers were: (1) were family members of the patients, (2) were designated by the patients as primary caregivers, (3) aged 18 years or older, and (4) provided informal care for patients without remuneration. Exclusion criteria were: (1) with significant cognitive or mental impairments; (2) had serious heart disease, cerebrovascular accident, or other diseases. If both the patient and the caregiver met the inclusion and exclusion criteria, they would be recruited for participating in this study.

### Sample size

According to the calculation formula of the cross-sectional survey, when α = 0.05, the corresponding Z_1-α/2_ is 1.96. Tan’s meta-analysis revealed that the prevalence of insomnia in hemodialysis patients was 46% (Tan et al., 2022). So p is set to 0.46 in this study. And the allowable error d is set to 0.1. The sample size required for this method is 96. In addition, considering when the sample size of the structural equation model is less than 200, the model parameter estimation is unstable, and the model’s statistical testing power is low. Therefore, 280 pairs of patients and caregivers were recruited for this study, and 259 valid questionnaires were recovered (if the questionnaire of the patient or caregiver was improperly (e.g., all items selected the same option), both of the questionnaires were excluded). The effective response rate was 92.5%.

$$N=\frac{Z_{1-\alpha /2}^{2}p\left(1-p\right)}{d^{2}}.$$

### Measures

The socio-demographic characteristics questionnaire was a self-made form. The investigation of patients included age, gender, educational level, employed status, marital status, family monthly income, and dialysis duration. The investigation of caregivers included age, gender, educational level, employed status, marital status, relationship with patient, and daily care duration. Daily care duration refers to the time that caregivers spend caring for patients, such as accompanying patients for treatment, helping patients with daily management, etc. It was filled in by caregivers according to the actual situation.

Perceived stress was measured by the Chinese Perceived Stress Scales (CPSS) (Yang, 2003). It is used to assess an individual’s stress level over the recent month. The scale has 14 items, including two dimensions: tension (7 items) and loss of control (7 items). Total scores range from 0 ~ 56, with higher scores indicating greater psychological stress. 0 ~ 28 indicates normal stress, 29 ~ 42 indicates high stress, and 43 ~ 56 indicates excessive stress. The Cronbach’s ɑ was 0.872 among the patients and 0.834 among the caregivers in this study. Confirmatory factor analysis showed the scale had good validity.

The Athens Insomnia Scale (AIS) (Soldatos et al., 2000) was used to assess insomnia. It consists of 8 items, each of which is rated from 0 to 3 on a scale of none to severe. If the respondent has the symptom at least 3 times a week during the past month, then select the appropriate option. The scale’s total score is the sum of the ratings for each item. A total score of <4 indicates that there is no sleep disorder, a total score of 4 to 6 indicates that there is suspected insomnia, and a total score of >6 indicates that there is insomnia. The Chinese version of AIS was discovered to have good validation (Chiang et al., 2009). The Cronbach’s ɑ was 0.820 among the patients and 0.891 among the caregivers in this study. Confirmatory factor analysis showed the scale had good validity.

The Perceived Social Support Scale (PSSS) was used to investigate the perceived social support of the respondents. It was translated into Chinese version by Huang (1996) in 1996 and demonstrated satisfactory reliability and validity. The simplified Chinese version of PSSS has 12 items and 3 dimensions: family support (items 3, 4, 8, 11), friends support (items 6, 7, 9, 12), and other support (item 1, 2, 5, 10). Each item was on a 7-point scale ranging from 1 (strongly disagree) to 7 (strongly agree). The total score was the sum of the scores of all items. A high score indicates stronger perceived social support. In this study, the Cronbach’s ɑ was 0.875 among the patients and 0.816 among the caregivers. Confirmatory factor analysis showed the scale had good validity.

### Procedures

Before the study, investigators accepted training to ensure that participants would receive consistent guidance. Before the study, the purpose, significance, and related concepts of the study were explained to the patients and caregivers. The questionnaire was distributed after written informed consent was obtained. Patients completed the questionnaires by themselves under the guidance of the investigators during the dialysis. The investigators assisted those who had trouble reading or writing the questionnaire. Caregivers completed the questionnaire in a separate room to avoid interaction. For those caregivers who were not at the site at that moment, the questionnaires would be completed by telephone (57 caregivers completed the questionnaires by telephone in this study). After completing the questionnaire, participants returned it to the investigators. Any information that could be used to identify the participants was anonymized.

### Statistical analysis

**Figure 1:**
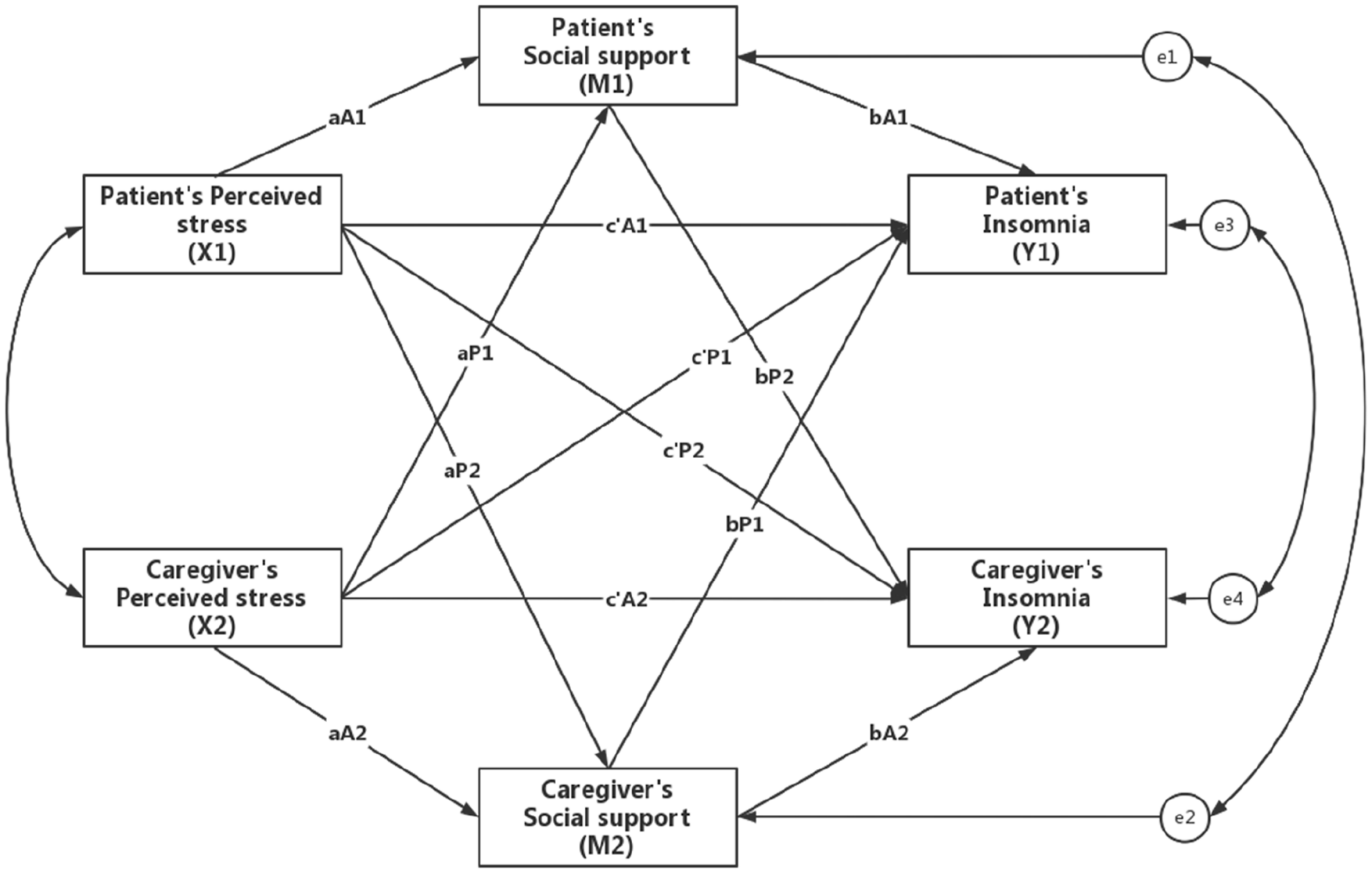
Hypothesized model. Notes: Actor effect: the effect of X1 to M1 (aA1), the effect of X2 to M2 (aA2), the effect of M1 to Y1 (bA1), the effect of M2 to Y2 (bA2), the effect of X1 to Y1 (c’A1), the effect of X2 to Y2 (c’A2). Partner effect: the effect of X1 to M2 (aP2), the effect of X2 to M1 (aP1), the effect of M1 to Y2 (bP2), the effect of M2 to Y1 (bP1), the effect of X1 to Y2 (c’P2), the effect of X2 to Y1 (c’P1).

Descriptive statistics were expressed as mean ± standard deviation (SD) for continuous variables and number (%) for categorical variables. Pearson’s correlation analysis was performed to examine the association between perceived stress, social support, and insomnia. This was used to test hypothesis 1, which stated that perceived stress and social support were correlated with insomnia both in hemodialysis patients and their family caregivers. The actor-partner interdependence mediation model (APIMeM) analysis was conducted to examine the effect of perceived stress on insomnia with the mediation of social support in patient-caregiver dyads (hypothesized model see Figure 1). This was used to test hypothesis 2, which proposed that social support mediated the relationship between perceived stress and insomnia in hemodialysis patient-caregiver dyads. Chi-squared/degrees of freedom ratios (*χ*^2^/df), root mean square error of approximation (RMSEA), comparative fit index (CFI), and Tucker-Lewis index (TLI) were used to examine the model fit (*χ*^2^/df < 3, RMSEA <0.08, CFI > 0.90, TLI > 0.90 indicate the model has a good fit) (Hair et al., 2019). The 95% confidence intervals (CI) were calculated based on 5,000 bootstrapping samples. Simple linear regression model was used to explore which socio-demographic variables may have effects on the model. SPSS 26.0 software and Mplus 8.3 program were used to analyse the data. *p* < 0.05 was considered statistically significant.

## Results

### Sample characteristics

The mean age of the hemodialysis patients and their family caregivers was 53.8 (*SD* = 15.5) and 53.5 (*SD* = 9.9) years. 66.0% of the patients and 37.8% of the caregivers were males. The most common educational level was junior high school or below in patients and senior high school in caregivers. Most of the patients were not working, and more than half of the caregivers were employed. 57.5% of the patients’ family monthly income was <3,000 yuan. 69.5% of the caregivers were patients’ spouses. Patients’ dialysis duration was 5.2 (*SD* = 4.2) years. Caregivers’ daily care duration was 8.6 (*SD* = 4.3) hours (Table 1).

**Table 1:** Demographic and disease-related characteristics (*n* = 259).

	Variables		*n* (%)	*M* ± *SD*
Patients	Age (years)			53.8 ± 15.5
	Gender	Male	171 (66.0)	
		Female	88 (34.0)	
	Educational level	Junior high school or below	110 (42.5)	
		Senior high school	93 (35.9)	
		Junior college or above	56 (21.6)	
	Employed status	Employed	37 (14.3)	
		Not working	222 (85.7)	
	Marital status	Married	212 (81.9)	
		Single or others	47 (18.1)	
	Family monthly income (RMB)	<3,000	149 (57.5)	
		3,000 ~ 5,000	75 (29.0)	
		>5,000	35 (13.5)	
	Dialysis duration (years)			5.2 ± 4.2
Caregivers	Age (years)			53.5 ± 9.9
	Gender	Male	98 (37.8)	
		Female	161 (62.2)	
	Educational level	Junior high school or below	82 (31.7)	
		Senior high school	100 (38.6)	
		Junior college or above	77 (29.7)	
	Employed status	Employed	124 (47.9)	
		Not working	135 (52.1)	
	Marital status	Married	238 (91.9)	
		Single or others	21 (8.1)	
	Relationship with patients	Spouses	180 (69.5)	
		children	32 (12.4)	
		parents	41 (15.8)	
		Other relatives	6 (2.3)	
	Daily care duration (hours)			8.6 ± 4.3

M, means; SD, standard deviation.

### Correlations between perceived stress, social support and insomnia

In hemodialysis patients, the mean score of perceived stress, social support, and insomnia was 31.91 (*SD* = 9.41), 47.18 (*SD* = 9.72) and 6.04 (*SD* = 3.47). 54.8% of the patients experienced high stress and 13.5% experienced excessive stress. The prevalence of insomnia among hemodialysis patients was 37.4 and 42.9% of patients were suspected insomnia. In caregivers, the mean score of perceived stress, social support, and insomnia was 30.92 (*SD* = 7.86), 48.56 (*SD* = 8.69) and 5.38 (*SD* = 2.27). 59.9% of the caregivers had high stress and 6.9% had excessive stress. The prevalence of insomnia among caregivers was 20.1 and 64.5% of caregivers experienced suspected insomnia (see Table 2).

**Table 2:** Correlation analysis of perceived stress, social support and insomnia (*n* = 259).

Variable	M	SD	1	2	3	4	5	6
1 Patient’s perceived stress	31.91	9.41	1					
2 Caregiver’s perceived stress	30.92	7.86	0.894^*^	1				
3 Patient’s social support	47.18	9.72	−0.613^*^	−0.577^*^	1			
4 Caregiver’s social support	48.56	8.69	−0.313^*^	−0.282^*^	0.425^*^	1		
5 Patient’s insomnia	6.04	3.47	0.839^*^	0.723^*^	−0.640^*^	−0.365^*^	1	
6 Caregiver’s insomnia	5.38	2.27	0.482^*^	0.387^*^	−0.370^*^	−0.638^*^	0.611^*^	1

M, mean; SD, standard deviation. *p < 0.05.

The correlation analysis results showed that both patient’s and caregiver’s perceived stress was positively correlated with insomnia (*r* = 0.387 to 0.839, *p* < 0.001), social support was negatively correlated with insomnia (*r* = −0.640 to-0.638, *p* < 0.001), and perceived stress was negatively correlated with social support (*r* = −0.613 to-0.282, *p* < 0.001). Hypothesis 1 was confirmed. Patient’s perceived stress and social support were correlated with caregiver’s insomnia (*r* = −0.370 to 0.482, *p* < 0.001), and caregiver’s perceived stress and social support were correlated with patient’s insomnia (*r* = −0.365 to 0.723, *p* < 0.001).

### Results of the APIMeM

**Figure 2:**
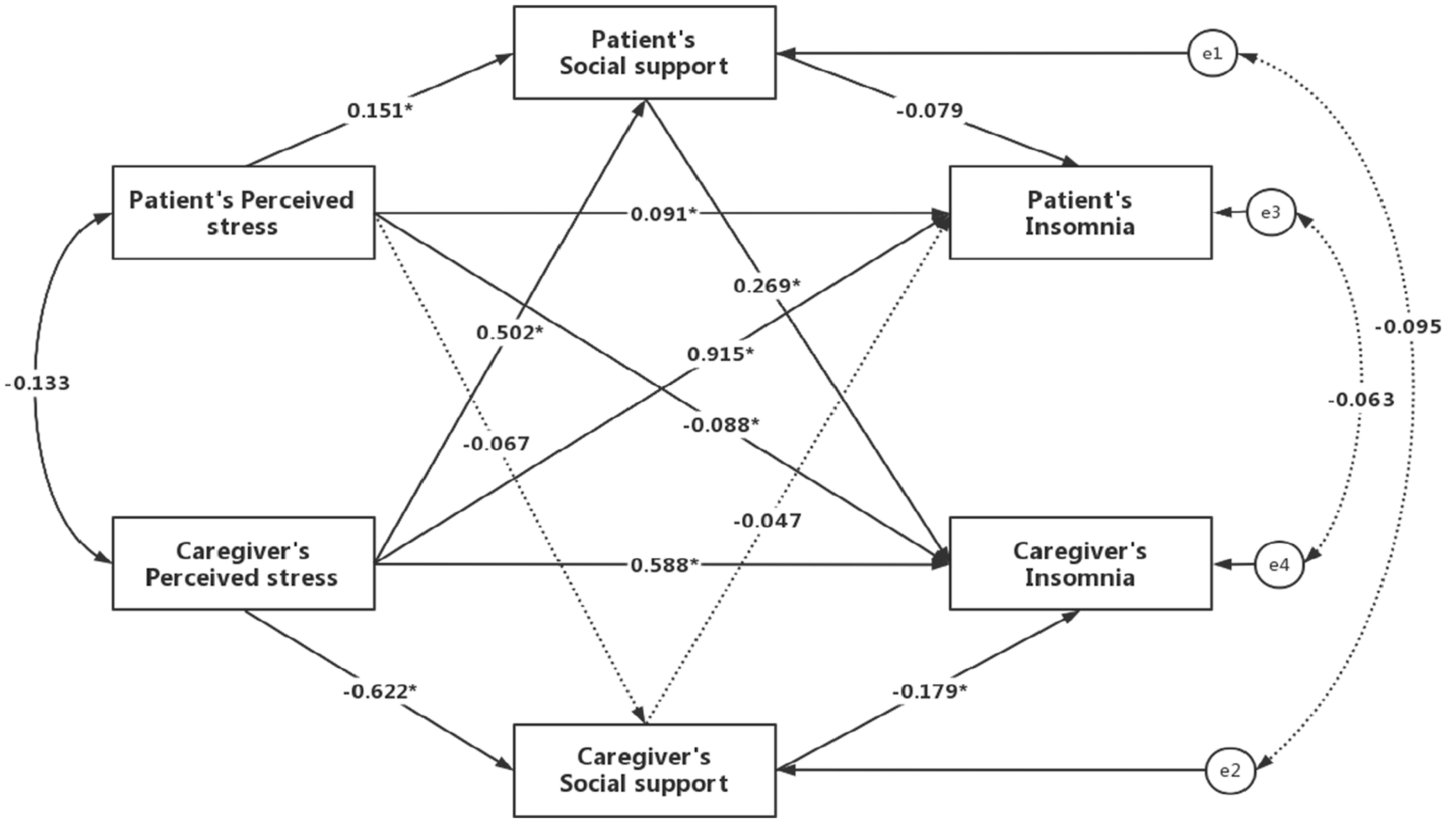
Actor-partner independence mediation model of perceived stress, social support, and insomnia in hemodialysis patients and their family caregivers. Solid lines represent significant paths, and dotted lines represent non-significant paths. *p < 0.05.

The results of the APIMeM between perceived stress, social support and insomnia in hemodialysis patients and their family caregivers were shown in Table 3. The data fit of the model was acceptable (*χ*2/df = 0.216, RMSEA = 0.064, CFI = 0.98, TLI = 0.94). The results of the simple linear regression model showed that the socio-demographic variables had no effects on perceived stress, social support, and insomnia in both hemodialysis patients and caregivers. Therefore, these socio-demographic variables were not included in the model. The standard coefficients of the model were shown in Figure 2.

**Table 3:** Total, direct, and indirect effects in the APIMeM (*n* = 259).

	*β*	*SE*	95%*CI*	*p*
Actor effect				
Patients				
Total effect	0.082	0.028	(0.016, 0.128)	**0.004**
Total indirect effect	−0.009	0.008	(−0.020, 0.009)	0.248
Specific indirect effect 1: X1-M1-Y1	−0.012	0.007	(−0.025, 0.003)	0.096
Specific indirect effect 2: X1-M2-Y1	0.003	0.004	(−0.002, 0.015)	0.401
Direct effect	0.091	0.029	(0.019, 0.134)	**0.001**
Caregivers				
Total effect	0.834	0.015	(0.825, 0.873)	**<0.001**
Total indirect effect	0.246	0.043	(0.263, 0.407)	**<0.001**
Specific indirect effect 1: X2-M1-Y2	0.135	0.028	(0.050, 0.126)	**<0.001**
Specific indirect effect 1: X2-M2-Y2	0.111	0.031	(0.191, 0.324)	**<0.001**
Direct effect	0.588	0.045	(0.433, 0.595)	**<0.001**
Partner effect				
Patients				
Total effect	0.905	0.021	(0.852, 0.927)	**<0.001**
Total indirect effect	−0.011	0.027	(−0.046, 0.068)	0.698
Specific indirect effect 1: X2-M1-Y1	−0.040	0.020	(−0.058, 0.011)	**0.046**
Specific indirect effect 1: X2-M2-Y1	0.029	0.023	(−0.026, 0.085)	0.204
Direct effect	0.915	0.035	(0.803, 0.953)	**<0.001**
Caregivers				
Total effect	−0.035	0.032	(−0.061, 0.066)	0.273
Total indirect effect	0.053	0.019	(0.028, 0.104)	**0.006**
Specific indirect effect 1: X1-M1-Y2	0.041	0.015	(0.013, 0.057)	**0.008**
Specific indirect effect 1: X1-M2-Y2	0.012	0.010	(0.005, 0.060)	0.229
Direct effect	−0.088	0.025	(−0.109, −0.008)	**0.001**

APIMeM, actor-partner interdependence mediation model; β, standardized estimate; SE, standard error; CI, confidence interval; X1, patient’s perceived stress; X2, caregiver’s perceived stress; M1, patient’s social support; M2, caregiver’s social support; Y1, patient’s insomnia; Y2, caregiver’s social support. The boldface indicates *p* < 0.05.

In the direct effects, patient’s and caregiver’s insomnia was both affected by their own perceived stress (*β* = 0.091, *p* = 0.001 and *β* = 0.588, *p* < 0.001) and social support (*β* = −0.079, *p* = 0.040 and *β* = −0.179, *p* < 0.001). Patient’s perceived stress (*β* = −0.088, *p* = 0.001) and social support (*β* = 0.269, *p* < 0.001) had an effect on caregiver’s insomnia. Patient’s insomnia was found to be significantly related to caregiver’s perceived stress (*β* = 0.915, *p* < 0.001), but not to caregiver’s social support (*β* = −0.047, *p* = 0.203). Patient’s social support was significantly correlated with patient’s and caregiver’s perceived stress (*β* = 0.151, *p* = 0.003 and *β* = 0.502, *p* < 0.001). However, caregiver’s social support was only significantly correlated with their own perceived stress (*β* = −0.622, *p* < 0.001). In addition, there was a significant correlation between the perceived stress of patients and caregivers (*β* = −0.133, *p* = 0.035).

In the indirect effects, caregiver’s perceived stress was associated with insomnia through the partial mediating effect of patient’s and caregiver’s social support (*β* = 0.135, *p* < 0.001 and *β* = 0.111, *p* < 0.001). The indirect effects of social support on patient’s perceived stress and insomnia were not significant, suggesting that insomnia was mainly caused by perceived stress in patients. The mediating effect of patient’s social support on the relationship between caregiver’s perceived stress and patient’s insomnia was statistically significant (*β* = −0.040, *p* = 0.046) in this study. However, as the *p* value is close to 0.05, the mediating effect needs to be further verified. Patient’s perceived stress was associated with caregiver’s insomnia through the partial mediating effect of patient’s social support (*β* = 0.041, *p* = 0.008). Hypothesis 2 was partially verified. The results indicated that caregiver’s sleep quality could be improved by reducing patient’s perceived stress and increasing patient’s social support.

## Discussion

In this study, the prevalence of insomnia in hemodialysis patients was 37.5%, which was similar to Rehman’s study (Rehman et al., 2020). The prevalence of insomnia among caregivers was 20.1%, which was higher than in community adults in China (Geng et al., 2021). Despite being a high priority for dialysis patients, sleep problems remain underdiagnosed and undertreated (De Silva et al., 2021). It is critical to pay closer attention to hemodialysis patient’s and caregiver’s insomnia. The mean score of perceived stress was 31.91 in hemodialysis patients, which was lower than Chen’s study (Chen et al., 2022). This may be due to the differences in the regions and populations. However, caregiver’s stress level was higher than that of Chinese healthcare workers (Wang et al., 2022). 68.3% of the patients and 66.8% of the caregivers had high or excessive stress. It is necessary to develop effective methods to reduce the stress level of hemodialysis patients and their family caregivers. In this study, the level of social support of caregivers was slightly higher than that of patients, both of which were at a medium level and needed to be further strengthened.

The results of this study showed that perceived stress is an important factor for hemodialysis patients and their family caregivers, which has a potential impact on both social support and insomnia. Previous studies have found that high levels of perceived stress can reduce social support for patients and caregivers, while also increasing the prevalence of insomnia (Shi et al., 2020; Kim and Cha, 2022). Affected by disease symptoms, complications, and long-term treatment, hemodialysis patients have high perceived stress levels, which leads to a variety of adverse outcomes, such as depression, insomnia, and decreased quality of life (Nadort et al., 2022). Caregivers of hemodialysis patients experience a high level of stress because of the difficult and stressful caregiving tasks, limitations in personal life, and uncertainty about the progression of the patient’s disease (Pinquart and Sörensen, 2003). This huge stress may lead to adverse mental health problems. Especially older caregivers, because of their health problems and functional limitations, may have a higher level of perceived stress (Koyanagi et al., 2018). Furthermore, we discovered that hemodialysis patient’s and caregiver’s perceived stress influenced each other in the APIMeM, implying that patients and caregivers should be viewed as a whole to comprehensively reduce the level of perceived stress.

Favorable social support helps patients cope better with the disease (Yang et al., 2019). Our study illustrated that social support partially mediated the association between perceived stress and insomnia in caregivers. However, perceived stress is the primary cause of insomnia in hemodialysis patients. This may be due to the limitations of the location and the number of samples. When perceived social support is lacking or limited, medical staff should plan appropriate interventions. The interventions should be tailored to the needs of the dyad and address various types of social support (De Maria et al., 2020). Family is the most important source of social support for patients (Iavarone et al., 2009). However, when family support is limited and cannot be improved, patients and caregivers can be encouraged to attend social activities and broaden the dyad’s social network (Russell et al., 2018).

In addition, the results showed that patient’s social support partially mediates patient’s perceived stress and caregiver’s insomnia. This suggests that increasing patient’s social support is effective in improving caregiver’s sleep quality. The mediating effect of patient’s social support on the relationship between caregiver’s perceived stress and patient’s insomnia was also statistically significant, but the *p* value was close to 0.05, and its effect needed to be further verified. However, this study did not find any dyadic interactions between caregiver’s social support and patient’s perceived stress and insomnia. Due to the scarcity of research on the effect of dyadic interaction between hemodialysis patients and their caregivers, the results of this study cannot be compared with other studies. The research in this area should be strengthened in the future.

The present study has some theoretical and practical implications. First of all, the results of this study expand the application of the dyadic illness management theory. The findings help researchers better understand the dyadic interaction between hemodialysis patients and their family caregivers. What’s more, the findings contribute to developing methods to reduce hemodialysis patient’s insomnia. Insomnia in hemodialysis patients requires regular screening for early intervention. Illness management is a dyadic process involving both the patient and the caregiver (Lyons and Lee, 2018). Medical staff can help hemodialysis patients and their caregivers reduce their perceived stress levels through relaxation training, meditation, or other methods (Alhawatmeh et al., 2022; Abu Maloh et al., 2023). Both social participation and close family relationships are important ways to boost social support. It’s worth noting that a distinctive feature of the dyadic approach to patient care is the focus on dyadic health (Uchmanowicz et al., 2022), rather than the individual patient or caregiver.

### Limitations and future studies

This study has several limitations. First, the study participants were recruited in two hospitals in Lanzhou, China. It was difficult to generalize the results to other areas due to different cultures and locations. Larger sample size and multi-center studies should be conducted to solve these problems in the future. Second, this was a cross-sectional study, and it did not account for dynamic changes in perceived stress, social support and insomnia in hemodialysis patients and caregivers. Longitudinal studies are required to investigate how the variables change over time. Third, the data were got from self-reported questionnaires, which may have recall bias and reporting bias. More precise measuring instruments and methods are suggested for future studies. Fourth, due to the limited number of investigators and the impact of the COVID-19 pandemic at the survey time, we did not investigate too much disease-related factors and residual confounders are possible. More influencing factors, such as dialysis frequency and complications in hemodialysis patients, should be considered.

## Conclusion

This study demonstrated the dyadic interactions of perceived stress, social support, and insomnia among hemodialysis patients and their family caregivers. More attention should be paid to the insomnia of hemodialysis patients as well as their family caregivers. Reasonable methods should be taken to improve their sleep quality. Reducing perceived stress levels and strengthening social support may be effective methods. However, patient-caregiver dyads should be taken as a whole to develop dyadic-based interventions.

## Data availability statement

Publicly available datasets were analyzed in this study. This data can be found here: The raw data supporting the conclusions of this article will be made available by the authors, without undue reservation.

## Ethics statement

The studies involving human participants were reviewed and approved by Ethical approval for the study was obtained from the Human Research Ethics Committee of Xi’an Jiaotong University Health Science Center, protocol number was 2022-1299. The patients/participants provided their written informed consent to participate in this study.

## Author contributions

YT, CN, and TL were responsible for the conception and design of the study. KZ, YH, and LF were in charge of data collection and study administration. All authors contributed to the article and approved the submitted version.

## Funding

This work was supported by the Department of Science & Technology of Shaanxi Province of China (Grand 2021SF-278 and Grand 2022SF-077).

## Conflict of interest

The authors declare that the research was conducted in the absence of any commercial or financial relationships that could be construed as a potential conflict of interest.

## Publisher’s note

All claims expressed in this article are solely those of the authors and do not necessarily represent those of their affiliated organizations, or those of the publisher, the editors and the reviewers. Any product that may be evaluated in this article, or claim that may be made by its manufacturer, is not guaranteed or endorsed by the publisher.
